# Whole lesion quantitative CT evaluation of renal cell carcinoma: differentiation of clear cell from papillary renal cell carcinoma

**DOI:** 10.1186/s40064-015-0823-z

**Published:** 2015-02-10

**Authors:** Frank Chen, Hannu Huhdanpaa, Bhushan Desai, Darryl Hwang, Steven Cen, Andy Sherrod, Jean-Christophe Bernhard, Mihir Desai, Inderbir Gill, Vinay Duddalwar

**Affiliations:** University of Southern California, 1500 San Pablo St, 2nd Floor Imaging, Los Angeles, CA 90033 USA; University of Southern California, 1510 San Pablo St, Suite 350, Los Angeles, CA 90033 USA; University of Southern California, 1520 San Pablo St, Suite 4600, Los Angeles, CA 90033 USA; University of Southern California, SSB 210B, Health Sciences Campus, Los Angeles, CA 90089 USA; University of Southern California, Health Sciences Campus, UNH 215, Los Angeles, CA 90089 USA; University of Southern California, 1441 Eastlake Avenue, NOR 7416, Los Angeles, CA 90033 USA; University of Southern California, 1441 Eastlake Avenue, NOR 2315, Los Angeles, CA 90033 USA

**Keywords:** Whole lesion enhancement parameters, Papillary renal cell carcinoma, Clear cell renal cell carcinoma, Histogram analysis

## Abstract

**Purpose:**

Clear cell renal cell carcinoma (ccRCC) is the most common subtype of renal cell cancer (RCC), followed by papillary RCC (pRCC). It is important to distinguish these two subtypes because of prognostic differences and possible changes in management, especially in cases undergoing active surveillance. The purpose of our study is to evaluate the use of voxel-based whole-lesion (WL) enhancement parameters on contrast enhanced computed tomography (CECT) to distinguish ccRCC from pRCC.

**Materials and methods:**

In this institutional review board-approved study, we retrospectively queried the surgical database for post nephrectomy patients who had pathology proven ccRCC or pRCC and who had preoperative multiphase CECT of the abdomen between June 2009 and June 2011. A total of 61 patients (46 with ccRCC and 15 with pRCC) who underwent robotic assisted partial nephrectomy for clinically localized disease were included in the study. Multiphase CT acquisitions were transferred to a dedicated three-dimensional workstation, and WL regions of interest were manually segmented. Voxel-based contrast enhancement values were collected from the lesion segmentation and displayed as a histogram. Mean and median enhancement and histogram distribution parameters skewness, kurtosis, standard deviation, and interquartile range were calculated for each lesion. Comparison between ccRCC and pRCC was made using each imaging parameter. For mean and median enhancement, which had a normal distribution, independent t-test was used. For histogram distribution parameters, which were not normally distributed, Wilcoxon rank sum test was used.

**Results:**

ccRCC had significantly higher mean and median whole WL enhancement (p < 0.01) compared to pRCC on arterial, nephrographic, and excretory phases. ccRCC had significantly higher interquartile range and standard deviation (p < 0.01) and significantly lower skewness (p < 0.01) compared to pRCC on arterial and nephrographic phases. ccRCC had significantly lower kurtosis compared to pRCC on only the arterial phase.

**Conclusion:**

Our study suggests that voxel-based WL enhancement parameters can be used as a quantitative tool to differentiate ccRCC from pRCC. Differentiating between the two main types of RCC would provide the patient and the treating physicians more information to formulate the initial approach to managing the patient’s renal cancer.

## Introduction

Renal cancer accounts for more than 2% of cancers in humans worldwide (Cho et al. 2011). In the United States, the annual incidence of renal cancer increased yearly by 1.6% over the past decade, with over 63,000 new cases in 2014 (SEER Cancer Statistics Review 1975). A majority of renal tumors are incidentally diagnosed on medical imaging and as a result, are often asymptomatic, small in size, and present at an earlier stage (Hock et al. 2002).

Clear cell renal cell carcinoma (ccRCC) is the most common subtype of renal cell cancer, which accounts for 60-65% of renal cell cancers, followed by papillary RCC (pRCC), which accounts for 13-15% of renal cell cancers. Several other types of renal cell carcinoma, such as chromophobe renal cell carcinoma, sarcomatous renal cell carcinoma, or oncocytic variants, for example, account for the remainder (Reuter 2006). Different tumor behavior and aggressiveness have been related to histologic subtypes. In the era of personalized medicine, this parameter has to be taken into account along with other well-established prognostic factors (Fuhrman grade, tumor size, and stage) (Ficarra et al. 2005; Fuhrman et al. 1982; Bretheau et al. 1995; Tsui et al. 2000; Cheville et al. 2001; Klatt et al. 2007; Lam et al. 2007; Delahunt 1998).

In the specific setting of small renal masses (<4 cm), active surveillance (AS) has progressively gained interest as an alternative to nephron sparing strategies (partial nephrectomy, ablative therapies). This option of care is particularly suitable for elderly or patients with significant comorbidities (Thompson et al. 2009; Kunkle et al. 2008; Chawla et al. 2006; Smaldone et al. 2012; Mason et al. 2011; Kouba et al. 2007; Pierorazio et al. 2012).

Nevertheless, AS requires strong patient commitment and acceptance. For this, any argument susceptible to influence decision-making and patient understanding without any added invasive procedure is highly valuable. pRCC have been reported to globally present a more favorable prognosis than ccRCC (Cheville et al. 2003; Teloken et al. 2009). Distinguishing between these two most common subtypes based on the CT scan only may enhance the quality of the information that is delivered to patients and their ability to make an informed consent.

ccRCC can often be distinguished from pRCC on multiphasic, multidetector computed tomography or multiphasic MRI based on qualitative evaluation, as ccRCC are more heterogeneous and enhance to a greater degree than pRCC. Quantitative methods of distinguishing ccRCC from pRCC on CT have relied on placement of regions of interest (ROI) on renal tumors to evaluate tumor enhancement characteristics. The ROI is placed on what is deemed the most avidly enhancing portion of the tumor (Young et al. 2013 May; Jung et al. 2012; Kim et al. 2002; Zhang et al. 2007; Sun et al. Mar 2009; Roy et al. 2007; Pedrosa et al. 2008; Bata et al. 2013; Cornelis et al. 2014; Pierorazio et al. 2013; Ruppert-Kohlmayr et al. 2004). This method is limited by sampling errors and interobserver variability in ROI placement. Whole lesion analysis of renal tumors would resolve these limitations.

The purpose of our study is to evaluate the use of voxel-based whole lesion enhancement parameters on contrast enhanced CT (CECT) to distinguish ccRCC from pRCC.

## Material and methods

### Patients

In this institutional review board (IRB)-approved, Health Insurance Portability and Accountability Act-compliant study, we retrospectively queried our IRB approved and prospectively maintained surgical database for post nephrectomy patients who had pathology proven ccRCC or pRCC and who had preoperative multiphase CECT of the abdomen between June 2009 and June 2011. A total of 61 patients who underwent robotic assisted partial nephrectomy for clinically localized disease were included in the study. Specimen analysis was performed by genitourinary specific pathologists, and histologic subtype was defined according to the World Health Organization 2004 classification (Eble et al. 2004).

### CT examination

All CT examinations were performed with a 64-detector row helical CT scanner (Brilliance, Philips Healthcare, CT). The CT scans were obtained during patient breath-holding with the following parameters: 120 kVp, variable tube current, slice thickness of 0.5 mm with reconstruction interval of 2 mm. Noncontrast, arterial, nephrographic, and excretory phase images of the abdomen were obtained. The pelvis was included on the nephrographic phase images. Approximately 100–150 mL of nonionic intravenous contrast material (Isovue 350; Bracco Imaging) dosed to weight was administered with a power injector at a rate of 5 mL/sec. Time delay to scanning for arterial phase images, nephrographic phase images, and excretory phase images were 25 sec, 90 sec, and 5 min respectively. Arterial phase images are obtained rather than corticomedullary phase images to create a true arterial map for surgical planning.

### CT analysis

**Figure 1:**
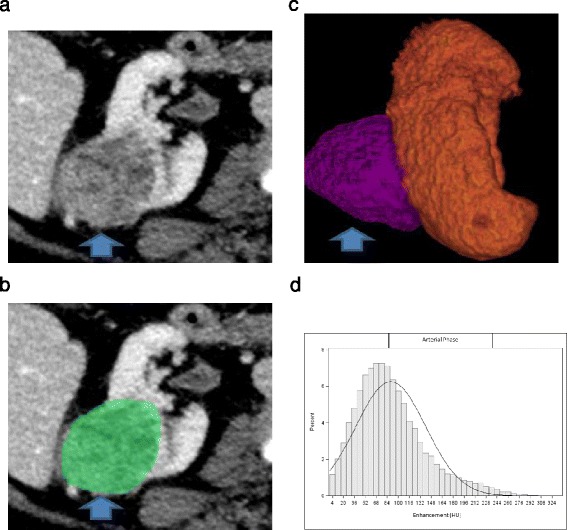
**A 60 y.o. with a mass (arrow) within the right kidney pathologically proven to represent ccRCC. (a)** On nephrographic phase CECT image, heterogeneously enhancing tumor is identified **(b)** The renal tumor was manually segmented **(c)** 3D ROI of the renal tumor was created **(d)** Histogram of whole lesion enhancement demonstrates relatively high mean and median arterial enhancement (89 HU and 81 HU respectively) and relatively high interquartile range and standard deviation (331 and 51 respectively).

**Figure 2:**
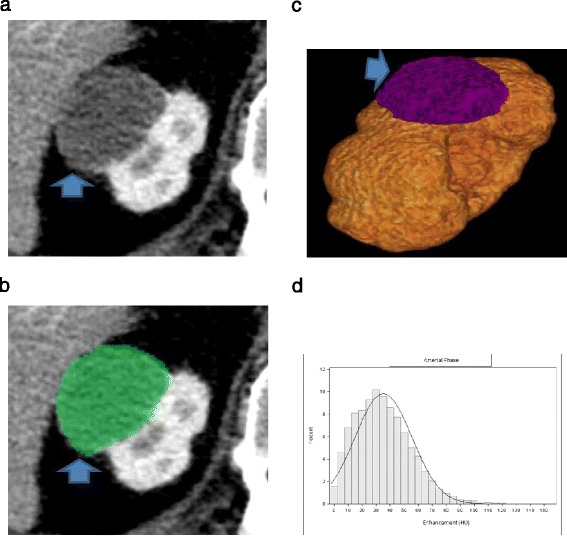
**A 67 y.o. with mass (arrow) within the right kidney pathologically proven to represent pRCC. (a)** On arterial phase CECT image, homogeneously enhancing tumor is identified **(b)** The renal tumor was manually segmented **(c)** 3D ROI of the renal tumor was created **(d)** Histogram of whole lesion enhancement demonstrates relatively low mean and median arterial enhancement (35 HU and 32 HU respectively) and relatively low interquartile range and standard deviation (155 and 20 respectively).

Multiphase CT acquisitions were transferred to our dedicated three-dimensional workstation (Synapse 3D; Fujifilm Medical Systems, Stamford, CT). Renal tumors were manually segmented in Synapse 3D as 3D ROIs. The kidney and tumor were segmented out in all phases to facilitate co-registration. The DICOM formatted CT images were converted into NIfTI volumes. DICOM keeps the images as individual slices while NIfTI treats images as multidimensional volumes. The series of images were then co-registered using normalized mutual information cost function implemented in Statistical Parametric Mapping software package. Custom MATLAB code was used to extract voxel data corresponding to the ROI. Contrast enhanced voxel enhancement values were displayed as a histogram (Figures 1 and 2). The histogram distribution parameters were computed using custom MATLAB analysis framework. The mean and median enhancement and histogram distribution parameters skewness, kurtosis, standard deviation, and interquartile range were computed for each lesion on all phases.

### Statistical analysis

Data distribution was examined using D’Agostino-Pearson test, Anderson-Darling test, and histogram based visual inspection. We used independent *t* test if the imaging parameter had a normal distribution or Wilcoxon rank sum if not a normal distribution. Receive operating characteristic (ROC) curve was used to estimate the prediction accuracy when predicting ccRCC versus pRCC using multiple imaging parameters. The candidate parameter was nominated from univariate testing. Candidate predictor selection criteria include 1) strongly associated tumor class 2) correlation coefficient with the parameter selected is less than 0.5. The gain of accuracy by adding additional parameter was tested using ROC contrast test. *P* values less than 0.05 were considered to indicate statistical significance.

## Results

### Patients

Of the 61 patients included in the study, 46 (75%) were male, and 15 (25%) were female. Forty-six patients had ccRCC, and 15 patients had pRCC. The mean age of patients with ccRCC was 61 years (range, 40–82 years), and the mean age of patients with pRCC was 62 years (range, 46–82 years). The mean tumor diameter was 3.1 cm for ccRCC (range, 0.7-5.5 cm) and 3.5 cm for pRCC (range, 2.0-6.0 cm).

### Enhancement

The mean and median enhancement of ccRCC and pRCC are summarized in Table 1. The distribution was considered as normal. The mean and median enhancement of ccRCC was significantly higher than that of pRCC in the arterial phase (mean enhancement, 93 HU vs 51 HU, p < 0.01; median enhancement, 91 HU vs 48 HU, p < 0.01), nephrographic phase (mean enhancement, 111 HU vs 76 HU, p < 0.01; median enhancement, 110 HU vs 72 HU, p < 0.01) and excretory phases (mean enhancement, 75 HU vs 60 HU, p < 0.01; median enhancement, 71 HU vs 57 HU, p < 0.01) (Figures 1 and 2).

**Table 1:** **Mean and median enhancement of ccRCC and pRCC**

**Enhancement**	**ccRCC**	**pRCC**	**p value**
Arterial – Mean	92.91 ± 35.59	50.6 ± 15.21	<0.01
Arterial – Median	92.24 ± 39.38	48.27 ± 15.1	<0.01
Nephrographic – Mean	110.66 ± 34.51	75.64 ± 18.28	<0.01
Nephrographic – Median	110.18 ± 38.59	72.13 ± 18.88	<0.01
Excretory – Mean	74.77 ± 16	60.33 ± 8.98	<0.01
Excretory – Median	71.28 ± 15.73	57.4 ± 8.68	<0.01

### Histogram distribution parameters

The histogram distribution parameters skewness, kurtosis, standard deviation, and interquartile range of ccRCC and pRCC are summarized in Table 2. The distribution was not normal, so that non-parametric statistics were used to compare the median. The histogram distribution parameters skewness and kurtosis of ccRCC were significantly lower than that of pRCC in the arterial phase (skewness, 0.29 vs 0.74, p < 0.01; kurtosis, −0.08 vs 2.03, p < 0.01) and nephrographic phase (skewness, 0.13 vs 1.06, p < 0.01; kurtosis, 0.11 vs 1.69, p < 0.01). The histogram distribution parameters standard deviation and interquartile range of ccRCC were significantly higher than that of pRCC in the arterial phase (standard deviation, 40 vs 24, p < 0.01; interquartile range, 263 vs 166, p < 0.01). Standard deviation and interquartile range of ccRCC were higher than that of pRCC in the nephrographic phase, but only standard deviation was significantly higher (standard deviation, 38 vs 32, p < 0.01; interquartile range, 250 vs 230, p = 0.12). The studied histogram distribution parameters of ccRCC were not significantly different than that of pRCC on excretory phase (Figures 1 and 2).

**Table 2:** **Histogram distribution parameters of ccRCC and pRCC**

**Histogram distribution parameter**	**ccRCC**	**pRCC**	**p value**
Arterial– Kurtosis (Q1, Q3)	−0.08 (−0.56, 0.94)	2.03 (0.34, 2.91)	<0.01
Skewness	0.29 (−0.12, 0.72)	0.74 (0.5, 1.31)	<0.01
Standard Deviation	40.25 (32.7, 51)	24.1 (19.4, 30.2)	<0.01
Interquartile Range	262.5 (210, 336)	166 (154, 258)	<0.01
Nephrographic – Kurtosis (Q1, Q3)	0.11 (−0.1, 0.6)	1.69 (0.39, 3.59)	<0.01
Skewness	0.13 (−0.33, 0.64)	1.06 (0.56, 1.37)	<0.01
Standard Deviation	38.3 (34.3, 44.9)	31.8 (27.4, 34.1)	<0.01
Interquartile Range	249.5 (226, 280)	230 (210, 266)	0.12
Excretory – Kurtosis (Q1, Q3)	2.15 (0.53, 16.21)	0.86 (0.19, 2.35)	0.22
Skewness	0.76 (0.04, 3.36)	0.61 (0.27, 1.42)	0.83
Standard Deviation	24.6 (20.7, 34.5)	24.5 (22.2, 25.9)	0.75
Interquartile Range	232.5 (159, 528)	163 (160, 184)	0.12

**Figure 3:**
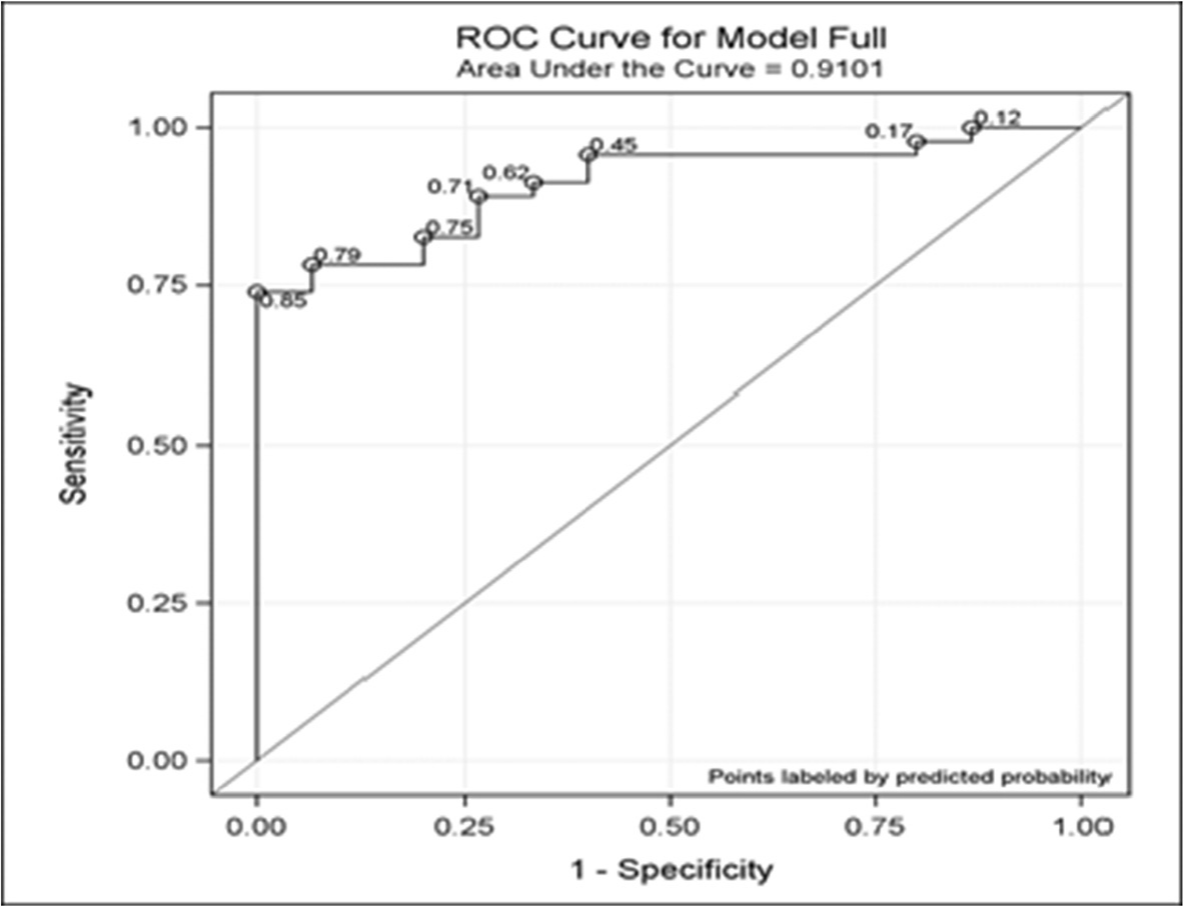
**ROC curve based on mean enhancement on arterial phase and interquartile range on nephrographic phase.** AUC of 0.91 with a 95% confidence interval (0.85-0.99).

The parameters selected for the final prediction model is mean enhancement on arterial phase and interquartile range on nephrographic phase. The correlation coefficient between these two parameters is −0.02 (p = 0.84). The ROC curve based on mean enhancement on arterial phase and interquartile range on nephrographic phase has an area under the curve (AUC) of 0.91 with a 95% confidence interval (0.85-0.99) (Figure 3). Mean enhancement on arterial phase was the main contributor to the prediction model. Adding interquartile range on nephrographic phase only improved 0.04 ± 0.04 in AUC.

## Discussion

It is important to distinguish ccRCC from pRCC because ccRCC is generally considered to have a worse prognosis and is treated differently than pRCC, especially as it pertains to targeted therapy in advanced disease (Schrader et al. 2008; Choueiri et al. 2008; Heng et al. 2010; Bellmunt and Dutcher 2013). For tumors that are diagnosed at an early stage, both cancers are treated similarly, but ccRCC tends to have a greater propensity for aggressive behavior and pRCC tends to have slower growth. Currently, if technically feasible, partial nephrectomy is considered the standard of care by the American Urological Association for the treatment of the clinical T1 renal mass. If partial nephrectomy is not technically feasible, radical nephrectomy is the alternate standard of care. Ablative techniques and active surveillance are considered options for patients who are high risk surgical candidates given major comorbidities or who wish to avoid surgery and are willing to assume the oncologic risks (Algorithm of Clinical Management of Clinical T1 Renal Mass 2009). Recent studies have shown that small renal masses which measure less than 3 cm have a slow growth rate and a low metastatic potential (Thompson et al. 2009; Kunkle et al. 2008; Chawla et al. 2006; Smaldone et al. 2012; Mason et al. 2011; Kouba et al. 2007; Pierorazio et al. 2012). These studies suggest that active surveillance may be an acceptable initial approach for the treatment of small renal masses, especially in patients who have major comorbidities. If pRCC can be reliably differentiated from ccRCC preoperatively, this would provide added information that can be used by the clinician to assist the patient in deciding between nephrectomy, ablative techniques, or active surveillance as the initial approach to treat the patient’s renal cancer. One method to differentiate ccRCC from pRCC is biopsy. However, biopsy techniques have added implications such as sampling error and introduce the risks of an invasive procedure (Volpe et al. 2007; Kümmerlin et al. 2008; Lebret et al. 2007; Schmidbauer et al. 2008). The less invasive technique to differentiate between ccRCC from pRCC is either CECT or MRI. Diffusion-weighted MRI has been shown to be potentially useful in distinguishing malignant and benign renal neoplasms (Mytsyk et al. 2014; Agnello et al. 2013).

On CECT, ccRCC is currently distinguished from pRCC based on qualitative assessment, which is fraught with subjectivity, or based on placement of ROIs on the most avidly enhancing portions of the tumor, which is limited by sampling error and interobserver variability. In this study, we sought to determine whether voxel-based whole lesion enhancement parameters on CECT can be used to distinguish ccRCC from pRCC. Whole lesion evaluation would eliminate sampling error and interobserver variability that limits ROI-based evaluation of renal tumors. In our study, we found that the median and mean whole lesion enhancement of ccRCC was significantly higher than that of pRCC on all postcontrast phases.

Whole lesion evaluation would also address the issue of tumor heterogeneity which some authorities believe could explain differential tumor behavior (Greller et al. 1996; Rickets and Linehan 2014). While the genetic heterogeneity of tumors has been studied, we propose whole lesion evaluation as a possible step in the quantification of tumor heterogeneity on imaging. In our study, we also compared the histogram distribution parameters kurtosis (degree of peakedness of a distribution), skewness (degree of asymmetry of a distribution), standard deviation, and interquartile range of ccRCC and pRCC. These histogram distribution parameters, especially interquartile range and standard deviation, are ways to evaluate variance within a data set. Since ccRCC is more heterogeneous than pRCC, its voxel-based data set would be expected to have greater variance (higher standard deviation and interquartile range, lower kurtosis) than the voxel-based data set of pRCC. As expected, in our study, we found that the skewness of ccRCC was significantly lower than that of pRCC, and the standard deviation and interquartile range of ccRCC are significantly higher than that of pRCC on arterial and nephrographic phases. Kurtosis of ccRCC was significantly lower than that of pRCC on only the arterial phase. To our knowledge, our study is the first study to evaluate the use of whole lesion histogram distribution parameters to distinguish ccRCC from pRCC on CECT. Chandarana et al. evaluated whole lesion histogram distribution parameters to discriminate ccRCC from pRCC on MRI and found that the skewness and kurtosis of ccRCC were significantly lower than that of pRCC on arterial phase. They did not compare the standard deviation and interquartile range of ccRCC and pRCC (Chandarana et al. 2012).

Our study had a few potential limitations. First, our study was a retrospective study. Second, we did not evaluate other subtypes of RCC, which have an even lower incidence than pRCC. Third, we did not differentiate the two subtypes (type 1 and type 2) of pRCC, which have different prognoses. Type 1 pRCC tends to have a much better prognosis than type 2 pRCC (Delahunt et al. 2001; Delahunt and Eble 1997). Fourth, whole lesion evaluation of renal tumors is technically more challenging than both qualitative assessment and ROI-based assessment of renal tumors, and as a result, may not be feasible in all clinical practice. It is also unclear whether whole lesion evaluation is more accurate than qualitative assessment and ROI-based assessment. Therefore, further investigation to directly compare whole lesion evaluation and ROI-based evaluation of renal cell carcinoma, both for accuracy and ease of use, is warranted.

We propose that whole lesion quantitative enhancement will serve as a more accurate baseline to monitor lesion growth in patients who are managed conservatively. It would also serve as a potential quantitative metric in monitoring the effects of chemotherapy, such as anti-angiogenic chemotherapy. The potential for multiparametric imaging evaluation using conventional standard of care imaging would provide the physician team more information. For patients undergoing biopsies, this technique may also provide more information regarding specific sampling sites.

In conclusion, our study suggests that voxel-based whole lesion enhancement parameters can be used as a quantitative tool to differentiate ccRCC from pRCC. Differentiating between the two main types of renal cell carcinoma would provide the patient and the team of physicians taking care of the patient more information when deciding whether the patient is to have the renal mass resected, ablated, or to undergo active surveillance.
